# Impact of Particle Size on the Physicochemical, Functional, and *In Vitro* Digestibility Properties of Fava Bean Flour and Bread

**DOI:** 10.3390/foods13182862

**Published:** 2024-09-10

**Authors:** Sunday J. Olakanmi, Digvir S. Jayas, Jitendra Paliwal, Rotimi E. Aluko

**Affiliations:** 1 Department of Biosystems Engineering, University of Manitoba, 75 Chancellors Circle, Winnipeg, MB R3T 5V6, Canada; olakanms@myumanitoba.ca (S.J.O.); j.paliwal@umanitoba.ca (J.P.); 2 President’s Office, University of Lethbridge, 4401 University Drive West, Lethbridge, AB T1K 3M4, Canada; 3 Department of Food and Human Nutritional Sciences, University of Manitoba, Winnipeg, MB R3T 2N2, Canada; rotimi.aluko@umanitoba.ca

**Keywords:** fava beans, *in vitro* digestion, particle size, flour milling, pulse flour, composite bread

## Abstract

Fava beans, renowned for their nutritional value and sustainable cultivation, are pivotal in various food applications. This study examined the implications of varying the particle size on the functional, physicochemical, and *in vitro* digestibility properties of fava bean flour. Fava bean was milled into 0.14, 0.50, and 1.0 mm particle sizes using a Ferkar multipurpose knife mill. Physicochemical analyses showed that the 0.14 mm flour had more starch damage, but higher protein and fat contents. Functionality assessments revealed that the finer particle sizes had better foaming properties, swelling power, and gelation behavior than the coarse particle size. Emulsion capacity showed that for all the pH conditions, 1.00 mm particle size flour had a significantly higher (*p* < 0.05) oil droplet size, while the 0.5 and 0.14 mm flours had smaller and similar oil droplet sizes. Moreover, *in vitro* digestibility assays resulted in improved starch digestion (*p* ˂ 0.05) with the increase in flour particle size. Varying the particle size of fava bean flour had less impact on the *in vitro* digestibility of the bread produced from wheat–fava bean composite flour, with an average of 84%. The findings underscore the critical role of particle size in tailoring fava bean flour for specific culinary purposes and nutritional considerations.

## 1. Introduction

Pulses (dried and edible grain legumes) belong to the plant family Leguminosae, with over 13,000 species and about 600 genera. The commonly consumed pulses include peas, dry beans, common beans, chickpeas, lentils, navy beans, pigeon peas, fava beans, and pinto beans [1,2,3,4]. These pulses are utilized either in whole or split forms for different food applications for human consumption and livestock feeds in different parts of the world. The reason for the global consumption of these grains is due to their several nutritional benefits for humans; for example, they are rich in proteins (20–35%), dietary fiber (15–20%), resistant starch, vitamins, and minerals, and they are also low in fats (<10%). In addition, many bioactive compounds with health benefits can be derived from pulses [1,2,5]. Considering the tremendous nutritional qualities of pulses, their regular consumption has been reported to offer numerous health benefits, e.g., the regulation of blood cholesterol levels, the control of diabetes, reductions in obesity risk factors, and the amelioration of gastrointestinal syndrome, colorectal cancer, and cardiovascular diseases. Hence, the food industry has made an increasing effort to use pulses or their derived ingredients to improve the nutrient profiles of food products [1,6,7].

Compared with lentils, common beans, and chickpeas, the fava bean (*Vicia faba* L.) (faba beans, broad beans, field beans, or horse beans) is considered an underutilized legume, especially in Western countries. The global production of fava beans is estimated at 5.7 million tons (Mt) compared to peas (14.6 Mt) and soybeans (353 Mt) [8]. In Canada, fava bean production between 2018 and 2023 ranged from 68,000 to 125,000 t, mostly used locally for livestock feeds (mostly hog and poultry) and feeding pets [9]. This underutilization is despite its nutritional and agronomical advantages over other pulse types and its ability to thrive in temperate environments, including Canada. Fava beans have a higher protein content (about 27.6% compared to common beans—22.17%, lentils—22.15%, and chickpeas—19.53%) and dietary fiber (about 25%) [8,10]. The protein contents of 11 fava bean varieties grown in three regions of western Canada between 2006 and 2007 varied between 27.5 and 32.4% [11]. In addition, fava beans are a rich source of bioactive compounds, including flavonoids and other phenolics, which possess excellent antioxidant properties [10]. Furthermore, fava beans are rich in essential micro and macronutrients that support the body’s growth and perform various physiological functions [10,12]. Fava beans are also gluten-free, non-GMO, and not a regulated allergen in Canada [13,14]. Like other legumes, fava beans have low glycemic indexes and are considered a less expensive source of plant protein for many countries, especially low-income countries [14]. One of the reasons for the low interest in fava beans, especially in human food, could be attributed to its antinutrients such as tannins, saponins, vicine, and convicine, with the latter two compounds being the major causes of favism [15]. However, with improved breeding approaches, low tannin [8] and vicine and convicine cultivars (e.g., Fabelle used for the current study) are now commercially available.

Like other pulses or cereals, fava beans are milled to produce flour suitable for different food purposes. These flours are commonly mixed with wheat flour to create a variety of wheat-based products, including bread, pasta, noodles, and their gluten-free counterparts. Upon the addition of water and subsequent processes like mixing and heating, these flours undergo different physicochemical changes, such as the gelatinization of starch, enzyme inactivation, and the denaturation of proteins, which make them suitable for different applications in food systems like binders, thickeners, gelling agents, and stabilizers [1]. The particle sizes of the flour play a crucial role in these techno-functional and physicochemical properties. Hence, these flour properties can be altered by varying the sieve opening sizes or adopting different milling methods [1,16,17,18,19].

Different studies have shown that modifying the pulse flours’ particle size affects their techno-functional and proximate properties. Pea flour with finer particle sizes has been reported to have lower starch gelatinization temperatures but improved pasting viscosities compared to coarse particle sizes. Also, the flours with smaller particle sizes had more starch, whereas their protein contents were insignificantly different from those with coarser particle sizes [17]. Also, a study has shown a corresponding decrease in the gelling strength with an increase in the particle size of barley and lentil flours [18]. Another study also showed a gradual increase in the starch content and a decrease in the amount of protein and ash of lentil flour milled with roller mills and sieved using 210, 149, 105, 74, and 63 µm screens as the particle sizes decreased [20]. In addition, soy flour with smaller particle sizes have been reported to contain increased lipid, protein, and ash contents compared with coarser particles [21]. In terms of digestibility, findings have shown that damaging the cell walls of starch granules increases the digestion rate because the accessibility of the digestive enzymes is enhanced [5]. The protein hydrolysis rate of red kidney beans was reported to be initially slower for undamaged cells than damaged cells, although similar hydrolysis levels for both were achieved with an extended digestion time [22]. The effects of different processing methods on the quality of protein have been explored for pinto beans [23] and black, fava, pinto, navy, red kidney, and navy beans [24], as well as green and red lentils [25]. Different operations, such as cooking and extrusion, produced comparable protein digestibility values; however, decreased rates of digestion were often observed after baking.

To increase the utilization of fava beans in the production of human foods, e.g., bread, snacks, pasta, and noodles, it is imperative to investigate the properties of the flour when milled into different particle sizes. Therefore, this research aimed to assess the implications of various particle sizes on the proximate, physicochemical, and techno-functional properties of fava bean flours. Also, the effect of the variation in particle size on starch and protein digestion of the flour was investigated. The effect on protein digestion in bread was investigated when wheat flour was mixed with fava bean flour at different ratios and particle sizes. A good comprehension of the effects of fava bean flour particle size would enable researchers and food processors to use a suitable size classification process to obtain specific nutritional and/or techno-functional characteristics.

## 2. Materials and Methods

### 2.1. Flour Sample

This study used the Fabelle fava bean variety obtained from Prairie Fava Ltd. (Glenboro, MB, Canada). This variety has low vicine (0.04%) and convicine (0.01%) antinutrient levels [14,26]. The seeds were dehulled, pre-broken, and milled into three different particle size fractions using 0.14 mm, 0.50 mm, and 1.0 mm screen apertures, denoting fine, medium, and coarse fractions, respectively. This variation in the particle size of the flour using different screen apertures was carried out to obtain flour with varied but controlled functionalities. Comprehensive details of the flour sample and milling procedure have been reported [27,28]. 

### 2.2. Proximate and Physicochemical Properties 

The AACC method 44-15.02 procedure was employed to evaluate the moisture content (%) of the flour samples. The samples (2–3 g) were dried in an oven set to 130 °C for 1 h [29]. The Soxhlet extraction technique was employed to evaluate the fat content of the flour sample [30]. The procedure of Williams et al. (1998) was followed to determine the protein content (*N* × 6.25, where *N* represents the nitrogen content) of the flour samples previously dried overnight at 103 °C using the LECO FP-828 (LECO Corporation, St Joseph, MI, USA). Drift corrections were carried out daily using ethylenediaminetetraacetic acid (EDTA) [31].

The total starch content (%) of the flour samples was assessed by following the AACCI-approved method 76-13.01 using the Megazyme K-TSTA kit (Megazyme; Wicklow, Ireland) [29]. The starch damage (%) was quantified by following the AACCI-approved method 76-31.01 using the Megazyme K-SDAM kit (Megazyme; Wicklow, Ireland) [29]. A Minolta CR-410 colorimeter (Konica Minolta Ltd., Mississauga, ON, Canada), equipped with a D65 illuminant, was used to evaluate the color properties of the flour samples. Following the AACCI-approved method 14-30.01, a slurry of the flour samples was prepared with water using a sample cup with specified flour weight, water volume, and mixing and resting times. After resting, the color features (i.e., CIE L*, a*, b* values) were determined from the bottom surface of the sample cup [29]. All additional chemicals and reagents met the standards for analytical grade. The analyses were executed in three replicates to account for data variance.

### 2.3. Particle Size Distribution

To evaluate the particle size distributions of the fava bean flour, laser diffraction using the Malvern Scirocco 2000 Mastersizer (Malvern Panalytical, a division of Spectris Canada Inc., Montreal, PQ, Canada) was employed. The results were reported as different particle size analysis curve (d) percentiles. At d(0.1), which represents the 10th percentile, 10% of the sample volume is composed of particles of the specified size (μm) or smaller. At d(0.5), which indicates the 50th percentile, 50% of the sample volume contains particles of the indicated size (μm) or smaller. At d(0.9), which denotes the 90th percentile, 90% of the sample volume is made up of particles of the stated size (μm) or smaller.

### 2.4. Functional Properties 

#### 2.4.1. Water and Oil Absorption/Holding Capacity

The water absorption capacity (WAC) of the flour samples was evaluated in accordance with the protocol outlined by Beuchat (1977) with some adjustments as explained by Maskus et al. (2016), and the data were represented as the amount of water (g) absorbed per g of the flour samples [32,33]. To assess the oil holding capacity (OHC) of the flour samples, a given mass of the flour sample was dispersed in canola oil to attain a final concentration of 60 mg/mL. The resulting mixture was then vortexed and held at room temperature for 30 min before centrifugation at 5600 rpm for 30 min. This step was followed by draining the excess oil in the resulting mixture by inverting the tubes, and the residues were reweighed. The OHC of the samples was given as the volume of oil (g) retained per g of the flour samples [34].

#### 2.4.2. Emulsion Ability and Stability

The procedure outlined by Osemwota et al. (2022) was followed to determine the emulsion formation and stability of the flour samples [34]. Using each of the following buffer solutions—0.1 M acetate (at pH 3 and 5), phosphate (at pH 7), and Tris-base (at pH 9)—flour slurries were prepared to obtain a 20 mg/mL final concentration. Next, 1 mL of pure canola oil was added to each solution. Emulsions were prepared by employing a Polytron PT 10-35 homogenizer (Kinematica AG, Lucerne, Switzerland) fitted with a 12 mm shaft. The water/oil mixture was homogenized using a centrifuge set to 20,000 rpm for 1 min. Using a Mastersizer 2000 particle size analyzer (Malvern Instruments Ltd., Malvern, UK), the average oil droplet size (*d*_3,2_) of the resulting emulsions was measured by dispersing it with distilled water. In a dropwise manner, each of the resulting emulsions was added to approximately 100 mL of distilled water in the small volume wet sample dispersion unit fitted to the Mastersizer (Hydro 2000S; Malvern Instruments Ltd., Malvern, UK), ensuring the desired obscuration level. Measurements were taken automatically in triplicate for each emulsion, with duplicates prepared for each flour sample. After taking the readings, the resulting emulsions were held undisturbed at room temperature for 30 min and the oil droplet size readings were taken again to evaluate how stable the formed emulsions were. The results obtained, with the inclusion of the oil droplet size of the emulsions (*d*_3,2_), were utilized to estimate the flour’s emulsifying capacity, whereas the formed emulsion’s stability (oil droplets taken after 30 min) was computed as

(1) $$Emulsion\;stability\left(\%\right)=\frac{Average\;oil\;droplet\;size\;measured\;at\;0\;\min}{Average\;oil\;droplet\;size\;measured\;after\;30\;\min}\times 100$$

#### 2.4.3. Foaming Capacity and Stability

To evaluate the foaming capacity of the flour samples, flour samples to obtain 10, 15, and 20 mg/mL final concentrations formulated with 0.1 M acetate (at pH 3 or 5), phosphate (at pH 7), or Tris-base (at pH 9) buffer solutions were dispersed in graduated centrifuge tubes (*V*_0_). A solution of the resulting flour mixtures was obtained by using the polytron PT 3100 homogenizer set to 20,000 rpm for 1 min and fitted with a 20 mm shaft (Kinematica AG, Lucerne, Switzerland). Then, the volume of the foam formed after homogenization (*V*_1_) was taken as the foam capacity (FC) of the flour samples, and the stability of the formed foam was recorded as the amount/volume of formed foam remaining after 30 min (*V*_2_) [34].

(2) $$Foam\;Capacity\;(\%)=\frac{V_{1}-V_{0}}{V_{0}}\times 100$$

(3) $$Foam\;Stability\;(\%)=\frac{V_{2}}{V_{1}}\times 100$$

where *V*_0_ is the volume obtained prior to whipping, *V*_1_ is the volume of the foam obtained post-whipping, and *V*_2_ is the foam volume remaining after 30 min.

#### 2.4.4. Water Solubility Index (WSI)

The procedure outlined by Gani et al. (2015) was employed to evaluate the WSI of the flours with some modifications [35]. About 2 g (named *M*_0_) of the fava bean flour was mixed with 40 mL of deionized water and vortexed for 1 min. Thereafter, the mixture was centrifuged at 1000 rpm for 15 min, and the clear liquid obtained was poured off. This process was then followed by inverting the centrifuge tubes on a dry paper towel for 5 min. Using a forced convection oven (Gallenkamp, Cambridge, UK), the sample residue was dried at 50 °C for 24 h and weighed (denoted as *M*_1_). The WSI of the sample was estimated as outlined below:

(4) $$WSI=\frac{M_{0}-M_{1}}{M_{0}}\times 100$$

where *M*_0_ is the initial weight of the flour sample and *M*_1_ is the weight of the flour residue obtained post-drying.

#### 2.4.5. Swelling Power

The procedure outlined by Gani et al. (2015) was employed to evaluate the swelling power of the flour samples with some modifications [35]. A mixture of the flour samples was obtained by adding about 1 g of the flour samples (denoted by *M*_0_) to 10 mL of distilled water in a centrifuge tube and heated in a shaking water bath preheated at 80 °C for 30 min. Once the heating process was completed, the formed suspension was subjected to centrifugation set to 1000 rpm for 15 min. Thereafter, the clear liquid (supernatant) was poured off, and then the weight of the resulting slurry, denoted as *M*_1_, was recorded. The following equation was used to calculate the swelling power:

(5) $$Swelling\;Power=\frac{M_{0}}{M_{1}}$$

where *M*_0_ denotes the initial weight of the flour samples and *M*_1_ denotes the weight of the flour paste once centrifugation was completed.

#### 2.4.6. Gel Formation Capacity

The gel formation capacity of the fava bean flours was evaluated by determining its least gelling concentration (LGC), as outlined by Osemwota et al. (2022) with minor alterations [34]. A stipulated amount of the flour samples was dispersed in 0.1 M phosphate buffer (at pH 7) at varying concentrations (2% to 40%, *w*/*v*, flour weight basis, at 2% intervals). The mixture was thoroughly mixed in a vortex for 5 min and subsequently transferred to a water bath (preheated to 95 °C) for 1 h. Thereafter, the heated sample was immediately cooled in running tap water and held in the refrigerator (maintained at 4 °C) for 14 h. After this, the tubes were brought out of the refrigerator and turned upside down to determine the minimum concentration of the flour at which the gel formed was retained in the tubes.

#### 2.4.7. Bulk Density

The bulk density of the flour samples (expressed as the weight (g) of flour per unit volume (g/mL^3^)) was determined following the procedure outlined by Ahmed et al. (2016) [20]. The analysis was performed in triplicate to account for data variability.

### 2.5. Pasting Properties

The Rapid Visco Analyzer (RVA) 4500 (Perten Instruments, Stockholm, Sweden) was used to assess the pasting properties of the flour samples by following the AACCI-approved method 76-21.01 (STD1; 13 min profile) [29]. The peak time, peak viscosity, hot paste viscosity, breakdown, setback, pasting temperature, and final viscosity were evaluated. The analysis was conducted thrice to take into account the data variance.

### 2.6. In Vitro Digestibility

The protocol of Hsu et al. (1977), also reported by Osemwota et al. (2022), was applied to evaluate the *in vitro* protein digestibility (IVPD) of the fava bean flour and bread samples [34,36]. A total of 30 mL of distilled water was used to dissolve about 600 mg of each flour sample and the pH of the mixture was changed to a pH of 8 by adding a 0.1 M NaOH solution. This was followed by preparing a solution containing three enzymes (3.1 mg chymotrypsin, 1.6 mg trypsin, and 1.3 mg peptidase per mL) and transferring to an ice water bath. This was followed by adding the freshly prepared enzyme solution to each sample mixture at a 1:10 *v*/*v* ratio. Throughout the analysis, the solution was kept at 37 °C and stirred continuously using a magnetic stirrer. The mixture’s pH was documented every 30 s for 10 min with the use of a pH meter. Duplicate analyses of each of the samples were carried out, and the *in vitro* protein digestibility was determined by utilizing the regression equation stipulated by Hsu et al. (1977), as outlined below:

(6) $$Protein\;digestibility\;\left(\%\right),\;Y\;=\;{210.46-18.10X}_{f}$$

where *X_f_* is the obtained pH for each of the sample mixtures after the 10 min digestion period.

The starch digestibility (%) of the fava bean flour was determined by following the AACC Method 32-40.01/AOAC Method 2002.02 using the Megazyme K-DSTRS kit (Digestible and Resistant Starch Kit—NEOGEN Corp., Megazyme; Wicklow, Ireland) [37]. About 1 g of the flour samples was maintained at 37 °C with a mixture comprising pancreatic α-amylase and amyloglucosidase (PAA/AMG) dissolved in maleate buffer (pH 6). Aliquots of the reaction solution were removed at three different times to account for the differences in the starch hydrolysis rate. A 1.0 mL aliquot was removed after 20 min to estimate the rapidly digestible starch (RDS) fractions. The slowly digestible starch (SDS) was the fraction of starch digested after 120 min minus the starch digested after 20 min (i.e., SDS = starch value at 120 min − starch value at 20 min). After 240 min, 1 mL and 4 mL of the stirring aliquots were taken to measure the total digestible starch (TDS) and resistant starch (RS), respectively. The aliquots were removed three times while the suspension was stirred and transferred to 20 mL of 50 mM acetic acid (to terminate the reaction). For the digestible starches, these solutions were thoroughly mixed, and 0.1 mL of aliquots was incubated with 0.1 mL of AMG (100 U/mL) to hydrolyze the remaining traces of maltose to glucose, which was measured with GOPOD reagent (glucose oxidase plus peroxidase and 4-aminoantipyrine). For the RS, the aliquot was mixed thoroughly with an equal volume of 95% *v*/*v* ethanol. Thereafter, the sample was centrifuged at 4000 rpm for 10 min, and the remaining pellet was rinsed using aqueous ethanol (50% *v*/*v*) to get rid of the excess glucose. The pellet was then dispersed in a cold 1.7 M NaOH solution to dissolve the RS. The solution was neutralized using 1.0 M sodium acetate buffer (at pH 3.8); the starch hydrolyzed to glucose with AMG (3300 U/mL), and the glucose determined using the GOPOD reagent. The digestible starches (RDS, SDS, and TDS; g/100 g sample) and RS were calculated as follows:

(7) $$Digestible\;Starch\;\left(\frac{g}{100\;g\;\mathrm{sample}}\right)=∆A\times F\times EV/W\times 0.0189$$

(8) $$Resistant\;Starch=∆A\times F\times \frac{EV}{W}\times FV\times 0.000225$$

where ∆*A* is the absorbance reaction taken alongside the blank reaction after 20 min for RDS; for SDS, absorbance was taken after 120 min; absorbance after 20 min and absorbance after 240 min were taken for TDS and RS, respectively. *F* denotes the conversion from absorbance to µg (the absorbance recorded for 100 µg of D-glucose in the GOPOD reaction is evaluated) [*F* denotes 100 (µg of D-glucose) divided by the GOPOD absorbance.

*EV* denotes the extraction volume (41).

*W* denotes the weight of the flour sample analyzed.

### 2.7. Bread Production

**Figure 1:**
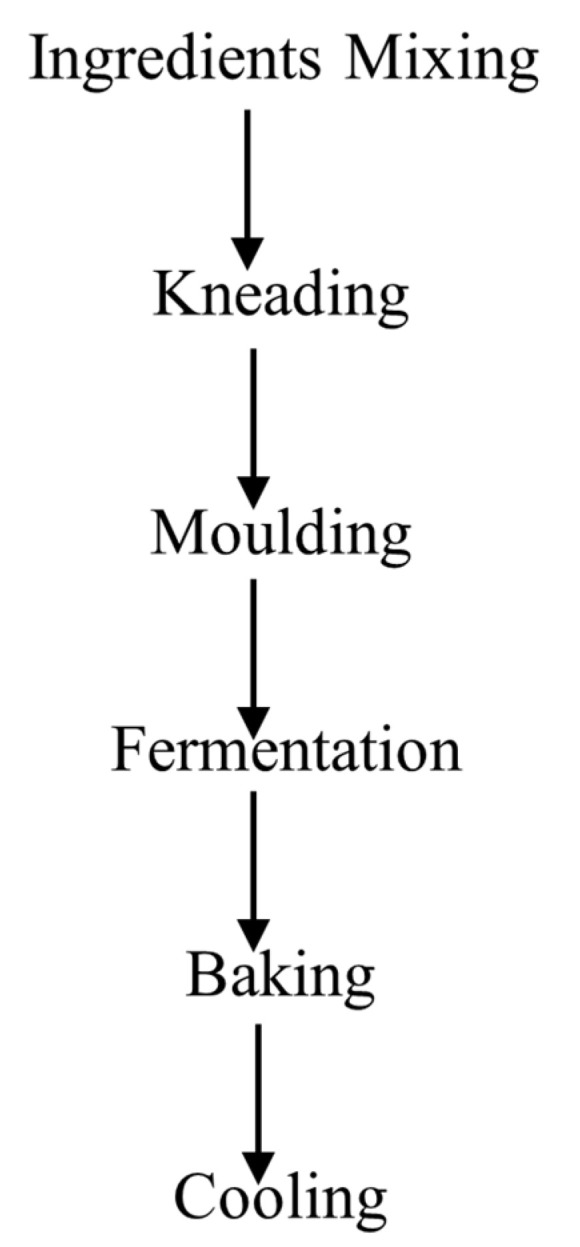
Test baking flowchart using the no-time dough method (NTD).

The no-time dough method (NTD) (Figure 1) was employed for test baking [27,28]. The wheat flour used for this study was obtained from Canada Western Red Spring wheat grade No. 1 seeds from the 2022 harvest. A wheat–fava bean flour blend was produced at 10, 20, and 30% fava bean flour substitution levels for the three particle sizes. These substitution levels were chosen because the blend above 30% resulted in dough with poor handling and viscoelastic properties unsuitable for bread production [28]. The blended flour samples, including 100% wheat flour (control), were kept in polyethylene bags and stored at a 15 °C temperature and 60% humidity until required for analysis. Bread was produced from the control sample (100% wheat flour) and the blended flour samples. A comprehensive procedure for test baking has been reported [27,28]. The breads produced were sliced and stored in a freezer maintained at −25 °C until further analyses.

### 2.8. Statistical Analysis

The data were analyzed using SPSS software (IBM Statistical Analysis Version 25.0). A one-way ANOVA was carried out to identify the significant differences between the flour samples, with the significance level set to *p* < 0.05. Duncan’s post hoc test was then applied to further evaluate the variations among the samples more precisely.

## 3. Results and Discussion

### 3.1. Proximate Composition and Physicochemical Properties 

Varying the particle size affected some physicochemical and proximate properties of the fava bean flour samples (Table 1). The moisture content among the three particle sizes was significantly different (*p* < 0.05), with the value increasing as the particle size increased. This trend could be due to the increased porosity in coarse particles compared to the finer ones. This increased porosity can provide more space for moisture to accumulate within the particles, resulting in higher overall moisture content. The results also showed that fava beans had a very high amount of protein (greater than 30%) and a low amount of fat (less than 2%) for all the particle sizes. These values are similar to the 30.4% and ~1% values reported for fava bean flour [1]. Similar trends were observed for the protein (%), starch damage (%, db.), and fat (%) contents of the flour. The smallest particle size had a higher value for these properties than the coarse size. For the fat content, varying the flour particle size did not result in appreciable differences (*p* > 0.05) among the samples. The flour with the largest particle size had significantly lower starch damage (%, db.) than the smaller ones. Different researchers have reported that pulse flours ground into fine particles exhibited more starch damage than coarse samples [17,33]. This could be attributed to a higher mechanical force needed to break the particles to go through the smaller screen [1,38]. Variations in the particle size did not produce a significant effect (*p* > 0.05) in the amount of total starch in the flour samples.

For the color characteristics, except the L* value for the 1.00 mm sample that was significantly different (*p* < 0.05) from the others, all the other color properties were unaffected by varying the fava bean flour particle size. Generally, varying the fava bean flour particle size had less impact on its physicochemical and proximate properties. Compared to other commonly consumed pulses like peas and lentils, fava beans have a substantially lower amount of starch and a higher amount of protein [1,19]. This result further demonstrates that fava beans are a superior and promising source of plant protein that can address the increasing demands for protein-rich ingredients in the industry.

### 3.2. Pasting Properties

The implication of fava bean flour particle size variation on pasting properties is presented in Table 2. The pasting property offers insights into the viscosity and the degree of retrogradation of the flour, which are important parameters that affect functionality. From the table, it can be deduced that there was a significant difference (*p* < 0.05) in the hot paste viscosity, peak viscosity, and final viscosity (cP) of the flour, with the flour with a 0.5 mm particle size having the highest value and the 1.00 mm flour having the least. The peak paste viscosity shows the capability of the flour to swell quickly before breaking down [39]. The results showed that the 0.5 mm flour particle size performed the best for these parameters, while the 1.0 mm flour performed the worst. The improved dispersibility of the fine flour particles could cause this. The increased surface area in fine particles allows for faster heat transfer, leading to faster and more even cooking. Gu et al. (2021) also reported that the 0.5 mm screen aperture yielded the best viscosity values for yellow pea flour in comparison to the 0.25 mm and 1.00 mm particle sizes [38].

For the breakdown, fewer variations were observed based on the particle size. This implies that the flours have comparable stability after hydration in water. However, the lower particle sizes (0.14 and 0.5 mm) would have been expected to have higher stability due to the higher starch damage than the 1.00 mm screen aperture. There were no significant differences (*p* > 0.05) in the peak time and pasting temperature values for the three particle sizes.

For the setback value (an indicator of temporary retrogradation), the smaller particle sizes (i.e., 0.14 and 0.5 mm) exhibited comparable behavior, which was significantly greater (*p* ˂ 0.05) than the 1.00 mm particle size. From this result, it can be concluded that the fava bean flour with a 0.5 mm particle size had the best pasting properties. The improved pasting properties of this particle size could be credited to the increased flour surface area for heat transfer and increased dispersibility of the particles. An important area to explore is understanding the effects of the nexus between the starch and protein content and the amylose–amylopectin ratio on the pulse flours’ pasting characteristics. This knowledge would help product developers adjust the flour’s composition to the desired levels to achieve baked products with the desired texture, structure, and crumb characteristics.

### 3.3. Particle Size Distribution

Table 3 shows the particle size distribution of the fava bean flour. A significant difference (*p* < 0.05) was observed in the particle size distribution of the flours. As anticipated, the flour milled using the smallest aperture (0.14 mm) exhibited the least particle sizes in contrast to the (0.5 mm) and (1.0 mm) screen apertures. The largest particle size flour produced significantly higher (*p* ˂ 0.05) d(0.1), d(0.5), d(0.9), and D[4,3] values. Also, the span and uniformity of the fine flour (0.14 mm) were significantly higher (*p* ˂ 0.05) compared to flours with larger particle sizes. Studies have shown that an increase in the average flour particle size results in a corresponding decline in its total surface-to-volume ratio. This leads to a greater diffusion and water penetration rate to the center of the particles for smaller particles than the larger ones [40].

### 3.4. Functional Properties

The capability of flour to retain oil and water is an essential characteristic in different baked goods, such as bread and meat formulations, as it influences the structure, texture, and mouthfeel of the end products [18,34]. Table 4 shows the fava bean flour’s water absorption capacity (WAC) as affected by varying particle size. The WAC of the 0.14 mm particle flour was significantly lower (*p* ˂ 0.05) in comparison to the 1.0 mm particle size. The lower WAC value of the smaller particle sizes could result from the further degradation of the starch granules due to the extended milling time. This decrease in the WAC can also be partly due to the reduced dietary fibers in the finer particle size flours [18]. This trend in the WAC of the flour agrees with the results reported by Bourré et al. (2019), who found a decrease in the WAC of yellow pea, navy pea, and red lentil flour milled using a Ferkar mill with decreasing particle size [17]. There was also a corresponding decrease in the WAC of pea, lentil, barley, and oat flours as the particle sizes decreased [18]. A more recent study showed that pea (yellow and green) flours milled using smaller screen size apertures had a lower WAC than the bigger particle size flours [41]. The result is, however, contrary to the general anticipation that the flour samples with finer particle sizes, having a larger surface area and more starch damage, would absorb more water than flours with coarser particle sizes. This further confirms that the WAC of flour depends on multiple factors that include the total starch, starch damage, protein and fiber content, and particle size. Thus, varying the particle size alone may not influence the WAC of flours [17,20,33]. 

The oil absorption capacity (OAC) (Table 4) spanned from 3.66 to 3.72 g/g of flour. The result of the OAC showed that the flour samples were not significantly different (*p* > 0.05) based on the particle size variations. Similar results have been reported that varying the particle sizes of navy bean, yellow pea, and red lentil flours milled with a Ferkar mill did not significantly affect its OAC [17]. In addition, varying the particle size of pea, lentil, barley, and oat flours did not affect their OAC [18]. Also, varying the particle size of pea (yellow and green) flours did not affect its OAC property [41]. However, some studies reported contrary results. Of note is a study that showed that Indian and Turkish lentil flours that have the finest particle size possess the lowest OAC value [20]. Another study has also reported that green lentils and yellow pea flours with larger particle sizes milled with a Ferkar mill had a higher OAC than those milled with a roller mill [42]. They attributed the high OAC value in the flours to the elevated protein levels in the flour, as the hydrophobic proteins possess a better cohesive strength to lipids [19,20,43].

The least gelling capacity (LGC) of the flour is the lowest concentration needed for the flour to form a successful non-sliding gel as a result of gravity when turned upside-down in a tube. Hence, flour with the lowest LGC has the highest gelling ability [34]. From Table 4, the gelling capacity of the finer particle sizes (i.e., 0.14 and 0.5 mm) was 14%, while that of the coarse particle size was 20%. The result implies that increasing the fava bean flour particle size reduces its gel-forming capacity. The improved gel-forming capacity of the finer flours could be associated with a higher surface area, resulting in better hydration rates. It has also been found that increasing pea, lentil, and fava bean flour particle sizes decreased their gel-forming capacity [1].

There was a gradual increase in the bulk density of the fava bean flour with a corresponding increase in the particle size. Also, the largest particle size possessed a significantly higher (*p* < 0.05) bulk density than the finer particle sizes. Factors influencing the bulk density include the particle size, moisture content, and other chemical constituents, as well as the method of determination. Also, the coarse particle size has a higher porosity value, contributing to the higher bulk density [44].

There was an insignificant difference (*p* > 0.05) in the swelling power of the flour based on the variation in the particle size. For the WSI, the finer particle size flour (0.14 and 0.50 mm) had similar WSIs, and the coarse particle had a significantly lower (*p* < 0.05) value. This difference in the WSI can be associated with the greater starch damage in the finer particle sizes (1.3%, db.) compared to the coarse particle size (1.0%, dry basis). The WSI provides knowledge about the strengths of the bonds in the starch granules and is a crucial way to predict the behavior of the flour during cooking [20,35].

**Figure 2:**
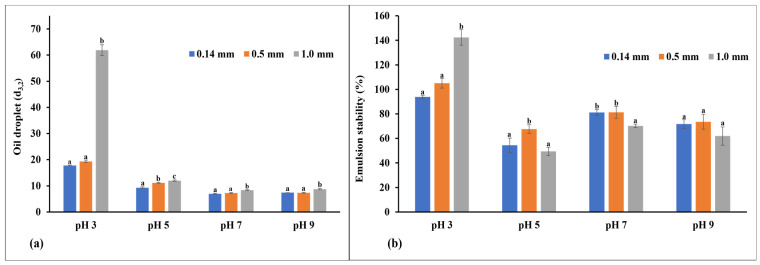
(**a**) Emulsion capacity and (**b**) emulsion stability. The data are expressed as mean ± SE (n = 10) for all measurements. For a given pH level, values with the same letters are not significantly different (*p* < 0.05), whereas values with different letters show significant differences (*p* < 0.05).

The ability of the flour to produce a continuous phase with vegetable oil is known as the emulsion capacity, while emulsion stability describes how stable the formed emulsions were after 30 min. In this study, the emulsion capacity and stability of the flour were examined at different pH levels. Studies have shown that the functionality of proteins is a function of both intrinsic (structure and molecular size) and extrinsic factors (i.e., its interactions with the processing conditions, including pH, thermal treatment, and ionic strength). Therefore, understanding the behavior of the flour under different pH conditions is essential for product manufacturers, especially those producing low-acid foods like salad dressings and mayonnaise [45]. Figure 2a,b show the emulsion capacity and stability of the flour at 20 mg/mL and different pH values. Figure 2a shows that the variations in the pH level affected the average oil droplet size (*d*_3,2_) of the flour, as the flour behaved differently under different pH conditions. At pH 3, the 1.00 mm particle size flour had the largest oil droplet size, while the smaller particle size flours had similar but lower oil droplet sizes. At pH 5, there was a significant increase (*p* < 0.05) in the oil droplet size with the corresponding increase in the flour particle size. At pH 7 and 9, the flour had a similar oil droplet size, with only the 1.00 mm particle size flour having a significantly higher (*p* < 0.05) oil droplet size. Overall, under all the pH conditions examined, the 1.00 mm particle size flour had a significantly higher (*p* < 0.05) oil droplet size, while the medium and fine flours had smaller and similar oil droplet sizes. The smaller oil droplet sizes with the smaller particle sizes imply they have a better emulsion-forming capacity. This could be due to their greater interaction with oil droplets as a result of their higher surface hydrophobicity [34]. However, it is possible that the improved surface area of the flours with smaller particle sizes was also a contributing factor by providing a larger binding area for the oil droplets in contrast to the coarse flour with a smaller surface area. This study confirms that modifying the particle size of fava bean flour has an effect on its emulsion capacity and its application in different food formulations.

The stability of the emulsion after allowing the emulsion to rest for 30 min undisturbed is shown in Figure 2b. At pH 3, the 0.14 mm and 0.5 mm flour exhibited a significantly reduced (*p* < 0.05) emulsion stability compared to the 1.00 mm particle size. At a pH 5, the medium particle size flour possessed a significantly higher (*p* < 0.05) stability than the fine and coarse particle sizes. However, at pH 7, the medium and fine particle size flours were more stable (*p* < 0.05) than the coarse particle size. At pH 9, the flours were stable in a similar pattern. The results suggest that varying the fava bean flour particle size affects emulsion stability. Overall, the fine and medium particle size flours tend to produce more stable emulsions, irrespective of the pH, which may be due to the smaller oil droplet sizes. Also, the smaller particle size flours had a more stable emulsion because of the increased surface area for protein adsorption, and the increased protein content in these smaller particle sizes ensures the stability of the emulsion interface [46,47,48].

**Figure 3:**
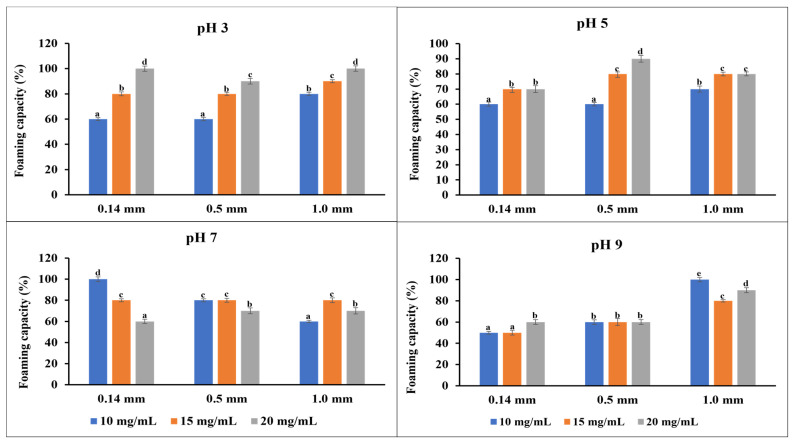
Foaming capacity of the flour at four different concentrations and four different pH levels. The data are presented as mean ± SE (n = 2) for all the measurements. For a given pH level, values with the same letters are not significantly different (*p* < 0.05), while those with different letters indicate significant differences (*p* < 0.05).

**Figure 4:**
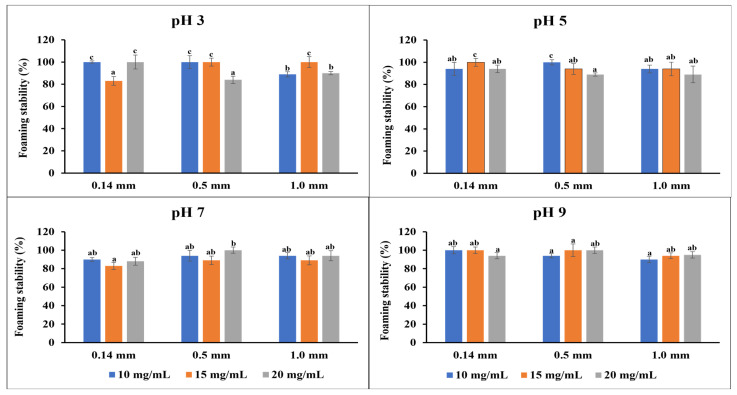
Foaming stability of the flour at four different concentrations and four different pH levels. The data are presented as mean ± SE (n = 2) for all the measurements. For a given pH level, values with the same letters are not significantly different (*p* < 0.05), while those with different letters indicate significant differences (*p* < 0.05).

The foaming capacity describes the volume increase of a sample after air is incorporated, while the foaming stability assesses the ability of the flour to retain air and prevent bubble collapse after 30 min. These properties are important in food products where aeration and overflow are necessary, such as cakes and ice cream, to enhance the texture and visual appeal of the products [34]. Figure 3 shows the foaming capacity of the flour samples at different concentrations and pH. At pH 3, the foaming capacity increased for each particle size as the flour concentration increased; a similar trend was observed at pH 5. However, at pH 7, a decline in the foaming capacity was observed with increasing flour concentration. At pH 9, lower foaming capacities were observed for the smaller particle size flour than the coarse particle size. Thus, the flour produces more foam in an acidic-to-neutral medium than in a basic medium. This could be caused by increased surface tension in the basic medium, making it more challenging for air bubbles to form and stabilize. Overall, increasing the flour concentration tends to significantly affect the foaming capacity of the flour than the variation in the particle size. These could be caused by increased viscosity, particle aggregation, and protein content of the solution as the flour concentration increases [49]. The foaming capacity of flour depends largely on the solubility of its proteins [34,50]. As shown in Figure 4, all the emulsions were mostly stable, irrespective of the variations in the particle size and flour concentration. The foam’s stability over an extended period is a desirable characteristic, especially when used as a whipping agent in food [44,51].

### 3.5. In Vitro Protein and Starch Digestion

Table 5 depicts the impacts of varying the particle size on the starch and protein digestibility properties of fava bean flour. It was found that increasing the particle size had an inverse relationship with the *in vitro* protein digestion of the flour. Though the fine particle size flour had the highest *in vitro* protein digestion, it was insignificantly different (*p* > 0.05) from other particle sizes. Tinus et al. (2012) also reported that the milling methods and particle size affect the digestibility of cowpea flour [52]. Byars et al. (2021) also reported an increase in navy bean flour’s *in vitro* protein digestion with decreased flour particle size [5].

For starch digestion, it was observed that varying the flour’s particle size produced a significant effect (*p* ˂ 0.05) on the starch digestibility property. The smaller particle size flour exhibited higher starch digestion, while the coarse particle size had more resistant starch. The starch digestibility of the fava bean flour is in the range of the values observed for different cultivars of lentil, pea, and chickpea flours. The improved digestibility of the finer particle sizes could be attributed to the increased starch damage [53]. Overall, the flour particle sizes impact *in vitro* protein and starch digestibility. Flours with finer particle sizes have a larger surface area, with the ability to produce an increased enzymatic action and improved digestibility. Coarser particles, in contrast, have reduced surface area, potentially leading to slower digestion. In addition, finer flour particles can absorb water more quickly, promoting starch gelatinization during cooking. The gelatinized starch is more readily broken down by enzymes, leading to speedy glucose release and improved digestibility. On the other hand, coarser flour particles may not fully gelatinize during cooking, causing slower starch digestion and a slower release of glucose. This can be beneficial for controlling blood sugar levels but might reduce overall nutrient absorption.

In summary, finer flour particles generally lead to an increased rate of nutrient digestion bioavailability but may also cause quicker spikes in blood glucose levels. Coarser flour particles can produce slower digestion rates, providing more sustained energy release and potentially benefiting gut health, but may reduce the immediate bioavailability of some nutrients. The particle size of flour influences the rate and extent of enzymatic reactions that break down proteins and starches during digestion [42,52,54,55,56].

### 3.6. In Vitro Protein Digestion of Fava Bean-Fortified Bread

Table 6 shows the effect of the fava bean flour substitution at varying particle sizes and levels of substitution on bread’s *in vitro* protein digestibility. Variations in the particle size and levels of substitution had less effects on the protein digestion of the bread. The complex processes involved in baking, including mixing, kneading, and baking (and the chemical processes involved), profoundly influence the bread’s digestibility [57]. Hence, merely varying the particle size of the pulse flour might not produce a pronounced effect on protein digestion. The values of the protein digestion of the bread were similar to the result (~84%) reported for bread with different formulations [58]. Overall, baking improves the protein digestibility of the bread compared to raw pulse flour. Different processing techniques, including baking, have been reported to positively affect the protein digestion of food products [59]. The baking process breaks down complex proteins into more digestible forms through denaturation, gelatinization, and other biochemical reactions. These processes help to improve the bioavailability of proteins by making them more easily digestible in the human digestive system.

In order to enhance the nutritional profiles of baked products using fava bean flour, it is imperative to study the effects of varying its particle sizes on the physicochemical, proximate, and functional properties. A good comprehension of the implication of varying particle size on the end product quality characteristics will allow product developers to make informed decisions on the flour particle size to achieve the desired end product quality [28]. Different studies have confirmed that varying the particle size of pulse flour affects the end product quality features. For example, it has been reported that varying the particle sizes impacted the textural properties of cookies produced from black soybean-fortified flour. They reported an increase in the hardness of the cookies as the flour particle size decreased. However, the fracturability, chewiness, and gluing properties of the cookies decreased with the decrease in particle size [60]. These textural features have an enormous impact on the sensory characteristics of the cookies. The crispiness, an important sensory feature, is negatively correlated with its fracturability, while the chewiness affects the taste and stickiness of the cookies [60,61]. It has also been reported that varying the particle size affected the color and specific volume of soy flour-fortified sponge cake. Thus, producing pulse flour-fortified baked products with the desired textural, nutritional, sensory, and handling characteristics demands a careful selection of flour particle sizes [21].

## 4. Conclusions

This study has elucidated the significant impact of particle size variations on the physicochemical properties, functional characteristics, and *in vitro* digestibility of fava bean flour. The results emphasize the dynamic nature of fava bean flour, indicating its potential for diverse applications in food product development based on the manipulation of particle size. It was found that particle size affects the techno-functional attributes, thereby influencing the flour’s behavior in food formulations and culinary needs.

Moreover, the study sheds light on the implication of particle size on the protein and starch digestibility, thereby providing insights into the bioavailability of the flour’s macronutrients. These findings underscore the importance of understanding the changes in fava bean flour when considering its use in various food products, such as bread. As the demand for sustainable and nutritious food sources continues to rise, the versatility of fava bean flour, as demonstrated through this research, presents an opportunity for innovative product development. A study has examined the impact of varying particle sizes and substitution levels on the dough rheology and baking characteristics of pan bread [28]. This research highlights the potential for further investigation into the effects of particle size variation in fava bean flour on other baked goods, such as cookies, cakes, doughnuts, muffins, and flatbreads. Additionally, exploring the cost implications of these flour blends and conducting environmental impact assessments could provide valuable insights to optimize the use of fava bean flour in diverse baked products while considering economic and environmental implications. By tailoring particle size to specific requirements, manufacturers can optimize the functionality and nutritional profile of fava bean flour-based products.

## Figures and Tables

**Table 1:** Proximate composition and physicochemical properties.

Flour Description	Moisture (%)	Protein (%)	Total Starch db. (%)	Starch Damage db. (%)	Fats (%)	Color Properties			
						L*	a*	b*	*WI*
0.14 mm fava bean	9.9 ± 0.0 ^a^	30.8 ± 0.1 ^b^	46.1 ± 1.1 ^a^	1.3 ± 0.03 ^b^	1.74 ± 0.28 ^a^	74.5 ± 0.1 ^a^	0.6 ± 0.01 ^a^	27.5 ± 0.2 ^a^	62.49 ± 0.21 ^a^
0.50 mm fava bean	10.3 ± 0.1 ^b^	30.5 ± 0.1 ^ab^	48.8 ± 0.9 ^a^	1.3 ± 0.03 ^b^	1.51 ± 0.11 ^a^	74.6 ± 0.1 ^a^	0.6 ± 0.05 ^a^	27.7 ± 0.1 ^a^	62.41 ± 0.14 ^a^
1.0 mm fava bean	11.1 ± 0.1 ^c^	30.4 ± 0.0 ^a^	45.2 ± 0.4 ^a^	1.0 ± 0.02 ^a^	1.44 ± 0.12 ^a^	75.2 ± 0.0 ^b^	0.5 ± 0.04 ^a^	27.4 ± 0.1 ^a^	63.00 ± 0.04 ^a^

*WI* is the whiteness index. The data are presented as mean ± SE (n = 3) for all the measurements. Values within a column sharing the same superscript letter indicate no significant difference (*p* < 0.05), whereas values with different superscripts are significantly distinct (*p* < 0.05). L* represents the whiteness or blackness of the flour sample (where 0 = black and 100 = white); a* indicates the level of redness (+) or greenness (−), and b* reflects the yellowness (+) or blueness (−) of the flour sample; db. denotes dry basis.

**Table 2:** Pasting properties.

Flour Description	Peak Viscosity (cP)	Hot Paste Viscosity (cP)	Breakdown (cP)	Final Viscosity (cP)	Setback (cP)	Peak Time (min)	Pasting Temp. (°C)
0.14 mm fava bean	1713 ± 54 ^b^	1720 ± 53 ^b^	−8 ± 0.5 ^ab^	3452 ± 29 ^b^	1732 ± 24 ^b^	7 ± 0.0 ^a^	78.3 ± 0.1 ^a^
0.50 mm fava bean	2040 ± 15 ^c^	2041 ± 14 ^c^	−1 ± 1.0 ^b^	3816 ± 20 ^c^	1775 ± 6 ^b^	7 ± 0.1 ^a^	76.7 ± 0.1 ^a^
1.0 mm fava bean	1299 ± 23 ^a^	1311 ± 26 ^a^	−12 ± 3.0 ^a^	2970 ± 37 ^a^	1659 ± 11 ^a^	7 ± 0.0 ^a^	77.4 ± 0.9 ^a^

The data are presented as mean ± SE (n = 3) for all the measurements. Values within a column sharing the same superscript letter indicate no significant difference (*p* < 0.05), whereas values with different superscripts are significantly distinct (*p* < 0.05); cP denotes centipoise.

**Table 3:** Particle size distribution **§**.

Flour Description	d(0.1)	d(0.5)	d(0.9)	D[4,3]	Span	Uniformity
0.14 mm fava bean	8.0 ± 0.5 ^a^	25.0 ± 0 ^a^	289.0 ± 8 ^b^	110.0 ± 3 ^b^	12.0 ± 0.4 ^c^	3.8 ± 0.14 ^c^
0.50 mm fava bean	9.0 ± 0.0 ^b^	34.0 ± 1 ^b^	247.0 ± 3 ^a^	88.0 ± 2 ^a^	7.0 ± 0.1 ^b^	2.1 ± 0.03 ^b^
1.0 mm fava bean	16.0 ± 0.0 ^c^	285.0 ± 1 ^c^	713.0 ± 8 ^c^	320.0 ± 3 ^c^	3.0 ± 0.02 ^a^	0.8 ± 0.01 ^a^

**§** d(0.1) represents the 10th percentile (µm), d(0.5) denotes the 50th percentile (µm), d(0.9) represents the 90th percentile (µm), and D[4,3] denotes the volume weighted mean (De Brouckere mean diameter) particles constituting the bulk of the sample volume. The data are presented as mean ± SE (n = 3) for all the measurements. Values within a column having the same superscript letter indicate no significant difference (*p* ˂ 0.05), whereas values with different superscript letters are significantly distinct (*p* ˂ 0.05).

**Table 4:** Functional properties **†**.

Flour Description	Swelling Power	WSI	LGC (%)	OAC (g/g)	WAC g/g	Bulk Density (g/mL^3^)
0.14 mm fava bean	4.52 ± 0.06 ^a^	37.69 ± 0.38 ^b^	14 ^a^	3.66 ± 0.21 ^a^	0.86 ± 0.01 ^a^	0.54 ± 0.01 ^a^
0.5 mm fava bean	4.59 ± 0.13 ^a^	38.51 ± 1.45 ^b^	14 ^a^	3.86 ± 0.18 ^a^	0.91 ± 0.02 ^ab^	0.55 ± 0.01 ^a^
1.0 mm fava bean	4.41 ± 0.04 ^a^	31.26 ± 0.88 ^a^	20 ^b^	3.72 ± 0.12 ^a^	0.96 ± 0.02 ^b^	0.76 ± 0.01 ^b^

**†** WSI is the water solubility index; LGC is the least gelation capacity; OAC is the oil absorption capacity; WAC is the water absorption capacity. The data are presented as mean ± SE (n = 3) for all the measurements. Values within a column having the same superscript letter indicate no significant difference (*p* ˂ 0.05), whereas values with different superscript letters are significantly distinct (*p* ˂ 0.05).

**Table 5:** *In vitro* protein and starch digestibility **§**.

Flour Description	IVPD (%)	RDS (%)	SDS (%)	TDS (%)	RS (%)
0.14 mm fava bean	75.95 ± 0.63 ^a^	6.03 ± 0.42 ^b^	31.10 ± 0.30 ^c^	38.43 ± 1.72 ^c^	7.68 ± 0.09 ^a^
0.5 mm fava bean	75.77 ± 0.09 ^a^	5.35 ± 0.47 ^b^	29.22 ± 0.34 ^b^	36.57 ± 1.01 ^b^	11.24 ± 0.31 ^b^
1.0 mm fava bean	75.59 ± 0.09 ^a^	3.20 ± 0.40 ^a^	27.15 ± 0.16 ^a^	30.35 ± 0.17 ^a^	14.80 ± 0.38 ^c^

**§** IVPD is the *in vitro* protein digestion; RDS is the rapidly digestible starch; SDS is the slowly digestible starch; TDS is the total digestible starch; RS is the resistant starch. The data are presented as mean ± SE (n = 3) for all the measurements. Values within a column having the same superscript letter indicate no significant difference (*p* ˂ 0.05), whereas values with different superscript letters are significantly distinct (*p* ˂ 0.05).

**Table 6:** *In vitro* protein digestibility of the fava bean-fortified bread.

Flour Description	Substitution Level	IVPD (%) †
Control	0	86.66 ± 1.09 ^b^
0.14 mm	10	82.22 ± 0.09 ^a^
	20	84.31 ± 1.26 ^ab^
	30	84.67 ± 1.27 ^ab^
0.50 mm	10	83.21 ± 2.18 ^ab^
	20	84.22 ± 0.63 ^ab^
	30	84.94 ± 0.09 ^ab^
1.00 mm	10	85.48 ± 0.09 ^ab^
	20	84.31 ± 0.37 ^ab^
	30	84.94 ± 1.00 ^ab^

**†** IVPD is the *in vitro* protein digestion. The data are presented as mean ± SE (n = 3) for all the measurements. Values having the same superscript letter indicate no significant difference (*p* ˂ 0.05), whereas values with different superscript letters are significantly distinct (*p* ˂ 0.05).

## Data Availability

The original contributions presented in the study are included in the article, further inquiries can be directed to the corresponding author.
